# Control of dengue larvae of *Aedes aegypti* and *Aedes albopictus* using the larvicidal bioactive compounds in different plant extracts and plant extract-mediated nanoparticles

**DOI:** 10.1186/s41182-024-00654-9

**Published:** 2024-12-18

**Authors:** Madhawa Pradeepa Nawarathne, Chathuranga Dharmarathne

**Affiliations:** 1 Postgraduate Institute of Science, University of Peradeniya, Peradeniya, Sri Lanka; 2 Department of Biological Sciences, Macquarie University, Sydney, NSW Australia

**Keywords:** *Aedes aegypti*, *Aedes albopictus*, Dengue, Essential oils, Encapsulation, Nanoparticles, Phytochemicals

## Abstract

**Background:**

Dengue is a devastating viral disease transmitted by mosquito vectors of *Aedes aegypti* and *Aedes albopictus*. Mosquito populations thrive in favourable breeding conditions, making mosquito control vital. Eliminating larval populations is the most effective method compared with other mosquito control methods. Synthetic chemicals such as organochlorine, organophosphate, carbamate and growth regulators are available for mosquito control, but their use is limited due to health and environmental concerns. Biologically synthesized insecticides are preferable to synthetic insecticides as they are eco-friendly, low cost, target-specific and less toxic for non-target organisms.

**Mainbody:**

Plant-derived bioassays are commonly used to control virally transmitted vectors, as plants contain bioactive compounds such as phytochemicals and essential oils that have high larvicidal efficacy against various mosquito vectors. In addition, nanomaterials are garnering attention in mosquito control due to their eco-friendliness, cost-effectiveness and safety. Commonly used nanomaterials include metal nanoparticles, such as silver nanoparticles, known for their potent larvicidal effect. Nanomaterials can be biologically synthesized through the combination with plant materials and encapsulation of bioactive compounds to maintain their stability and efficacy.

**Conclusion:**

Various plant species and parts, as well as plant-derived nanoparticles, show diverse larvicidal activities against *Aedes* mosquitos. Among these, plant-mediated nanoparticles demonstrate excellent larvicidal properties against mosquito larvae, including *Aedes* species.

## Introduction

Mosquitoes are vectors of several destructive viral diseases to humans. ZIKA, dengue, chikungunya, yellow fever, malaria and Japanese encephalitis are major viral infections that are transmitted by vectors [1]. Among the vector-borne diseases, dengue is considered one of the most dangerous, and is transmitted by *Aedes* mosquitoes such as *Aedes aegypti* and *Aedes albopictus* [2, 3] so vector control is crucial. Viruses have the ability to reproduce rapidly and accumulate mutations thereby quickly adapting to changing environments and gaining resistance to environmental controls. Consequently, vaccines are often ineffective for controlling viral diseases. With urbanization and environmental shifts, including alterations in temperature, humidity, sanitation facilities and water supply, the mosquito population has seen a significant surge, particularly in polluted areas where vector-borne diseases are prevalent. Polluted environments offer favourable conditions for mosquito breeding [4].

Mosquito control techniques include eliminating larval habitats, reducing life span of adult mosquitoes, biological control using larval predators and reduction and prevention of human–mosquito contact. Among these methods, exterminating the larval population is much easier than others since the larvae are aquatic. It is also a safer and more targeted method as the targets are confined to small areas [5]. Scientists have been actively engaged in the development of insecticides and synthetic agents to combat mosquito-borne diseases. Nowadays, a wide array of synthetic chemicals, such as organophosphates, organochlorines, carbamates, pyrethroids, and growth inhibitors are utilized for mosquito control [6]. However, their application comes with various drawbacks. These include high environmental risks, harm to non-target organisms, the development of resistance against chemical insecticides, adverse effects on human health, biological magnification, ecological sustainability concerns and prolonged persistence in the environment [5]. Therefore, there is a pressing need for novel technologies to address these limitations by introducing environment-friendly materials and innovative methods.

Plants serve as valuable sources of bioactive compounds including various phytochemicals and essential oils that can eliminate mosquito larvae without causing harm to other organisms or the environment. Plant roots, leaves, bark, stems, flowers, fruits and seeds are abundant reservoirs of these bioactive compounds [6]. With the development of nanotechnology, nanoparticles are widely used for various applications since they possess unique optical properties and are characterized by their eco-friendliness, effectiveness, affordability, non-toxicity, safety in handling, high sensitivity and selectivity. Furthermore, the advent of nanotechnology has garnered significant attention for controlling vector-borne diseases. Consequently, plant-mediated nanoparticles can exhibit excellent larvicidal activities against mosquito larvae. Among these, silver nanoparticles demonstrate potent larvicidal activity against various mosquito vectors with minimal or no adverse effects on non-target species [6, 7]. Plant materials play crucial roles as capping and reducing agents in nanoparticle synthesis. These plant-derived nanomaterials offer cost-effectiveness, eco-friendliness and swift action on the target. Moreover, their synthesis does not necessitate the use of toxic chemicals, high pressure, energy nor temperature [7]. This review highlights the effectiveness of plants and plant extract-mediated nanomaterials in combating dengue larvae.

## Evolution of insecticides against dengue vectors

Dengue became an epidemic with the increase of mosquito populations, making control challenging. In the past, dengue control methods relied on pyrethroid insecticides, reducing adult mosquito populations or organophosphates, such as temephos, thus eliminating *Aedes* larvae. DDT is an organochlorine that has been commonly used for mosquito control. In addition, insect growth regulators such as diflubenzuron are used to kill mosquitoes by disrupting chitin synthesis, changing mosquito metabolism, altering target sites that bind enzymes such as acetylcholinesterase enzymes and penetration into the body [8]. However, these chemicals produce toxic effects against aquatic and other non-target organisms. Therefore, biologically based insecticides are being developed as they offer many advantages, including being target-specific and cost-effective, along with minima impacts on non-target organisms. As an example, bacterial insecticides are produced using *Bacillus thuringensis* are a safe and efficient method. In addition, fungal and plant-based assays are widely synthesized for mosquito control [9].

## Larvicidal properties of plants

Plants have larvicidal properties due to the presence of phytochemicals that are secondary metabolites and serve as defense mechanisms by foraging herbivores [10]. Common phytochemicals are alkaloids, steroids, terpenes, flavonoids and phenolic compounds, all of which may have medicinal, pesticidal or larvicidal properties. The efficacy of phytochemicals as larvicides depends on plant species, plant components, extraction methodology, the polarity of the solvent used for the extraction, mosquito species and geographic origin of the plants [11]. These different phytochemicals are responded to in different ways [1, 10, 11]. As an example, alkaloids contain nitrogenous compounds that exhibit larvicidal potential at low concentrations by affecting acetylcholinesterase or sodium channels, as this inhibits the acetylcholinesterase activity responsible for terminating nerve impulse transmission through the synaptic pathways. Alkaloids have the ability to kill mosquito larvae by disrupting their life cycle and directly influencing the nervous system. Moreover, they can degrade cell membranes and act as mitochondrial poisons and stomach poisons [11, 12]. The most probable larval death is due to interacting with the cuticle membranes of the larvae disarranging the membrane [11]. Essential oils have neurotoxic effects on insects by inhibiting the acetylcholinesterase enzyme and blocking receptors of octopamine and GABA-gated chloride channels [12]. Flavonoids show inhibitory effects on the acetylcholinesterase and respiratory system of insects [11].

## Larvicidal plant extracts

Gutierrez demonstrated the effectiveness of phytochemicals against *Aedes* larvae. Stem, bark and leaf extracts of *Jatropha curcas, Tinospora rumphii* and *Citrus grandis* were used to determine larvicidal activity against *Aedes aegypti*. Alkaloids, steroids, flavonoids, tannins, saponins, anthraquinone and Cyanogenic glycosides were all identified as major bioactive compounds and they possess larvicidal properties. Larvicidal activity was assessed using a concentration series (20, 40, and 60 mg/mL) of methanolic plant extracts. The highest mortality was observed at 60 mg/mL for *T. rumphii*, likely due to its high phytochemical content. Larvae mortality rates of 90% and 93% were recorded after 24 and 48 h of exposure, respectively. *Jatropha curcas* leaf extract showed the least mortality due to the absence of saponins and tannins. However, all plant parts showed significant larval mortality and that increased with increase of the concentrations of plant extract and the number of phytochemicals that denote the larvicidal activity [5].

AhbiRami et al. investigated larvicidal activity of various plant parts: flowers, leaves, and stems of *Ipomoea cairica* (railway creeper) against third-instar larvae of *Aedes albopictus* and *Aedes aegypti* using methanol and acetone as the extraction solvents. Concentration series ranging from 10 to 450 ppm of crude plant extracts were utilized to determine larvicidal efficacy. While exposure to low concentrations (10 ppm) showed no mortality, exposure to high concentrations (450 ppm) resulted in the highest mortality rates (100% larvae mortality after 24 h). Furthermore, the researchers observed that *Aedes aegypti* exhibited greater susceptibility compared to *Aedes albopictus.* Among the three plant parts tests, leaf extracts demonstrated the highest larval mortality, followed by flowers and stems. Additionally, acetone extract of all plant parts exhibited higher mortality rates compared with methanol extracts. Acetone extract of *Ipomoea cairica* leaves displayed the most effective larvicidal activity [2].

Raveen et al. analysed the insecticidal potential of crude leaf extracts of *Alternanthera sessilis, Amaranthus dubius, Sesbania grandiflora* and *Solanum nigrum* against larvae of *Aedes aegypti*. The larvicidal potential was tested for each crude extract that was extracted from different solvent systems: hexane, butanol, propanol and ethanol. Among them, the ethanol extracts showed the highest larvicidal activity with 34.12, 51.16, 91.17, and 130.37 mg/L LC_50_ values for *Solanum nigrum, Sesbania grandiflora, Alternanthera sessilis and Amaranthus dubius*, respectively. *Solanum nigrum* exhibited the highest larval mortality (100%) due to the lowest LC_50_ value. Thus, results revealed that larval mortality depends on the plant species and plant parts. In addition, larvicidal activity relies on the concentration of plant extract, age and the solvent used in extraction [1].

Some plant roots contain phytochemicals that can be used as eco-friendly insecticides. As an example, Ali et al. investigated plant extracts from root and aerial parts of chicory (*Cichorium intybus*) and wormwood (*Artemisia absinthium*) against malaria, dengue and filariasis vectors. The larvicidal potential is determined using petroleum ether, chloroform, ethyl acetate, and methanol extracts from roots and aerial parts of chicory and wormwood against fourth instar larvae of mosquito vectors after 12 and 24 h exposures. Concentration series (200, 100, 50, 25, 12.5 ppm) of root and aerial part plant extracts were prepared by dissolving them in dimethyl sulfoxide (DMSO) and larvae mortality recorded. The highest larvicidal activity was shown in methanol extracts c/f other extracts. In addition, root extracts showed the highest mortality (lowest LD_50_) compared with aerial parts. While no mortality was observed in the control sample, the root extract of *A. absinthium* exhibited the highest larvicidal activity (LD_50_ = 59.37 ppm) compared with the root extract of *C. intybus* (LD_50_ = 66.16 ppm) in methanol. Thus, methanol extracts of *A. absinthium* and *C. intybus* can be used as effective vector biological insecticides against malaria, dengue and filariasis vectors [13].

### Larvicidal potency of plant essential oils against *Aedes* larvae

Plant essential oils are widely used for various diseases in plants. They are complex mixtures of many single molecules, derived from terpenes and their oxygenated compounds. Balasubramani et al. focused on the biological activity of essential oils extracted from *Artemisia vulgaris* (common mugwort) against third and fourth stage larvae of *Aedes aegypti* at different concentrations. Many volatile compounds are associated with these leaf essential oils which have insecticidal properties. Essential oils are extracted from hydro-distillation of dry leaves and mainly contain terpenoids, notably camphor, β-caryophyllene, α-humulene and β-caryophyllene oxide. The highest concentration (100 ppm) of plant extract showed the highest larval mortality with 6.87 and 4.28 ppm respective LC_50_ values for third and fourth stage *Aedes* larvae. These compounds interact with larval cells and destroy them. Therefore, the leaf essential oil of *Artemisia vulgaris* can be used for the development of highly effective insecticides against dengue vectors without any harmful effects on humans and other non-target species [14].

The larvicidal activity of leaf essential oils of *Heracleum sprengelianum* was investigated against *Anopheles subpictus* (malaria vector), *Aedes albopictus* (dengue vector) and *Culex tritaeniorhynchus* (Japanese encephalitis vector). Govindarajan and Benalli identified two main constituents that are present in high concentrations: lavandulyl acetate (17.85%) and bicyclogermacrene (12.9%). The bioassay was tested using third-instar larvae of the above vectors. As a result, significant larvicidal activities were exhibited with 33.4, 37.5 and 40.9 μg/mL LC_50_ values against *A. subpictus, A. albopictus* and *C. tritaeniorhynchus* larvae, respectively. Compared with bicyclogermacrene, lavandulyl acetate showed the highest larval mortality with the lowest LC_50_ values. Together lavandulyl acetate and bicyclogermacrene showed 3- to 7-fold larvicidal activity compared with the essential oil in the *Heracleum sprengelianum* against *Anopheles*, *Aedes,* and *Culex* larvae. Thus, the larvicidal activity of lavandulyl acetate and bicyclogermacrene can be used for preparation of inexpensive, target-specific, effective biological insecticides against dengue larvae without any effect on non-target species [15].

Isolation of *Cunninghamia konishii* (China-fir) wood extract exhibits larvicidal activity. Cheng et al. investigated the larvicidal activities of wood and leaf essential oils and ethanolic extract of *C. konishii* against *A. albopictus* and *A. aegypti* at different concentrations. While wood essential oils showed 100% larval mortality at 400 and 200 μg/ml for *A. aegypti* and *A. albopictus,* 95% larval mortality was shown for leaf essential oils. Highest effectiveness was exhibited in wood essential oils compared with the leaf essential oils against *A. albopictus* and *A. aegypti*. However, ethanolic extracts of wood and leaf did not show significant larvicidal activity. The wood essential oils of *C. konishii* can be used for the synthesis of inexpensive and environmentally safe biological insecticides [16].

### Larvicidal potency of marine plants against *Aedes* larvae

**Fig. 1:**
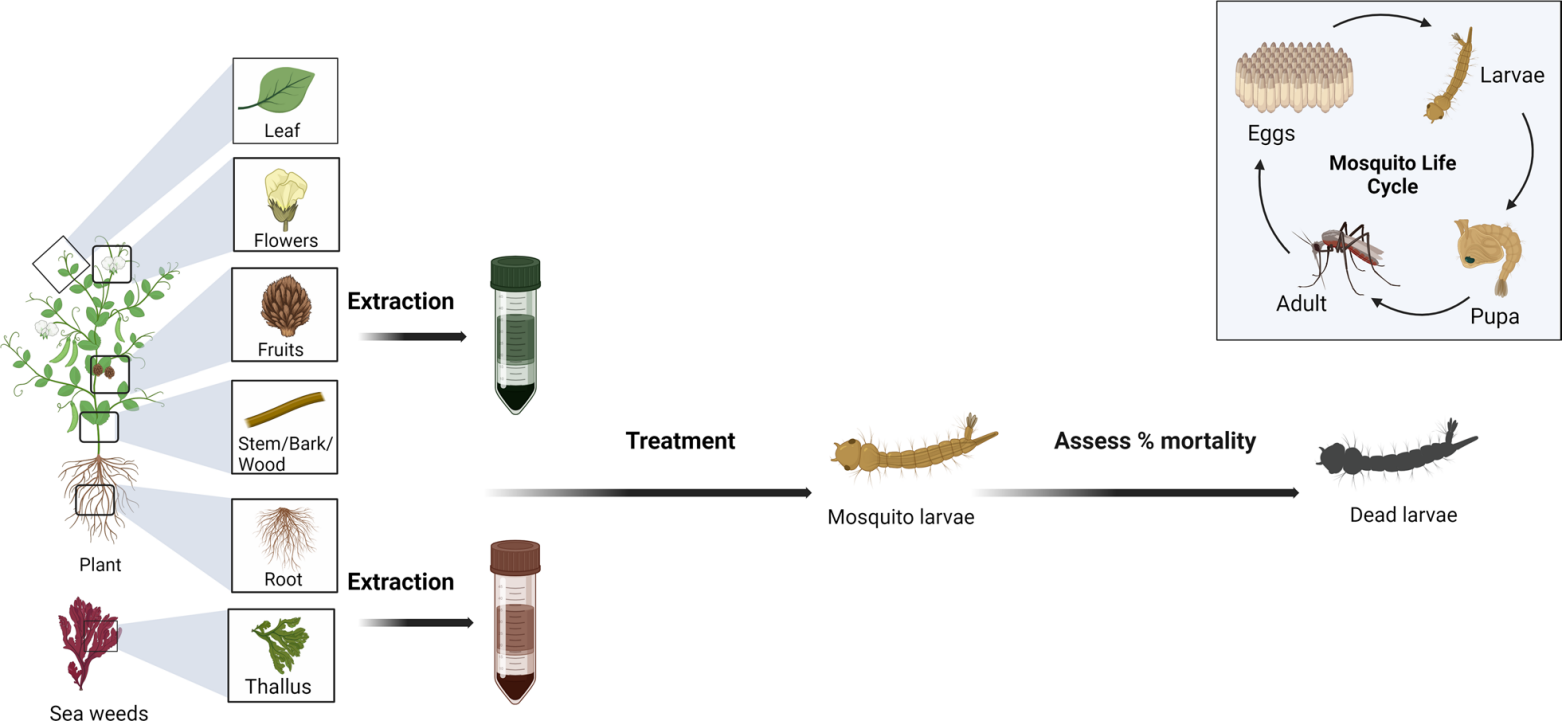
Process for the assessment of larvicidal activity of different extracts

Red and brown seaweeds are rich sources of bioactive compounds that have larvicidal activity [17–19]. Bianco et al. (2013), investigated the larvicidal activity of 15 extracts of red, brown and green seaweeds against dengue larvae. Among them, *Canistrocarpus cervicornis* (Kutzing)*, Laurencia dendroidea* (a red alga)*, Hypnea musciformis* (Crozier weed), and *Chaetomorpha antennina* (a green alga) extracts achieved more than 50% larval mortality at 300 ppm concentration for the fourth instar of *A. aegypti*. The strongest larvicidal activity was obtained from *L. dendroidea* extract. Phytochemicals were extracted using n-hexane, dichloromethane, ethyl acetate and methanol. These bioassays were applied to the fourth instar larvae of *A. aegypti* and the resultant crude extracts showed 100% larval mortality at 50 ppm. Moreover, n-hexane solvent extract represented the strongest larvicidal activity at 10 ppm (LC_50_ = 10.7 ppm) as seaweed extracts contain halogenated terpenes called elatol and they are influenced by the nervous system of *Aedes* larvae [17]. In addition, the larvicidal activity of extracts of *Ulva lactuca* (sea lettuce), *Caulerpa racemosa* (sea grapes), *Sargassum microcystum* (brown algae), *Caulerpa scalpelliformis* (scalpel green seaweed), *Gracilaria corticata* (Irish moss)*, Turbinaria decurrens* (triangular sea bell)*, Turbinaria conoides* (sea bell)*,* and *Caulerpa taxifolia* (killer algae) were investigated against *Anopheles stephensi*, *Aedes aegypti,* and *Culex quinquefasciatus*. Among these seaweed extracts, *Caulerpa racemosa* showed the highest larvicidal activity with the lowest LD_50_ values; 0.055, 0.0675, and 0.0661 μg/mL for respective larval species. Phytochemical screening revealed that *Caulerpa racemosa* contains a high content of carbohydrates, terpenoids, phenols, proteins and sugars compared with other alga extracts [18]. Table 1 shows the larvicidal activity of plant materials which are discussed in this review while Fig. 1 summarizes the experimental process of larvicidal bioassay.

**Table 1:** Overview on discussed larvicidal activity of different plant extracts in this review

Plant species	Family name	Plant part	Vector	%Mortality	LD_50_ value		References
					After 24 h	After 48 h	
*Jatropha curcas*	Euphorbiaceae	Leaf	*Aedes aegypti*	38.33	88 mg/mL	43 mg/mL	[5]
		Bark		73.33	53 mg/mL	44 mg/mL	
*Tinospora rumphii*	Menispermaceae	Leaf		90.00	16 mg/mL	10 mg/mL	
		Stem		86.67	33 mg/mL	23 mg/mL	
*Citrus grandis*	Rutaceae	Leaf		66.67	34 mg/mL	11 mg/mL	
		Bark		80.00	32 mg/mL	19 mg/mL	
*Ipomoea cairica*	Convolvulaceae	Leaf	*Aedes albopictus*		105.59 ppm	–	[2]
		Flower		100.00	132.47 ppm	–	
		Stem			145.79 ppm	–	
		Leaf	*A. aegypti*		101.94 ppm	–	
		Flower			105.53 ppm	–	
		Stem			132.94 ppm	–	
*Alternanthera sessilis*	Amaranthaceae	Leaf	*A. aegypti*	50.00	54.79 mg/L	–	[1]
*Amaranthus dubius*	Amaranthaceae			60.00	130.37 mg/L	–	
*Sesbania grandiflora*	Fabaceae			70.00	51.16 mg/L	–	
*Solanum nigrum*	Solanaceae			100.00	34.12 mg/L	–	
*Cichorium intybus*	Asteraceae	Root	*A. aegypti*	-	40.15 ppm	–	[13]
		Aerial		-	43.25 ppm	–	
*Artemisia absinthium*	Asteraceae	Root		-	41.41 ppm	–	
		Aerial		-	62.92 ppm	–	
*Cunninghamia konishii*	Cupressaceae	Wood	*A. aegypti*	100.00	85.70 ppm	–	[16]
			*A. albopictus*		189.5 ppm	–	
		Leaf essential oils	*A. aegypti*	98.10	91.70 ppm	–	
			*A. albopictus*		194.40 ppm	–	
*Artemisia vulgaris*	Asteraceae	Leaf essential oils	*A. aegypti (3rd stage)*	97.28	6.87 mg/mL	–	[14]
			*A. aegypti (4th stage)*	98.81	4.269 mg/mL	–	
*Heracleum sprengelianum*	Apiaceae	Leaf essential oils	*A. albopictus*	97.80	37.5 ppm	–	[15]
*Laurencia dendroidea*	Rhodomelaceae	Sea weeds	*A. aegypti*	100.00	10.7 ppm	–	[17]
*Caulerpa racemosa*	Caulerpaceae	Sea weeds	*A. aegypti*	100.00	67.5 ppm	–	[18]

## Synthesis and usage of nanomaterials against mosquito larvae

Nanoparticles can serve as larvicides for mosquito control, synthesized through physical, chemical and biological means. However, chemically synthesized nanoparticles often involve toxic chemicals and generate hazardous by-products, limiting their usefulness. Biologically synthesized nanoparticles, such as those mediated by plants and microorganisms, are safer and eco-friendly. When considering the synthesis of plant-mediated nanoparticles, plant extracts can be obtained from either the whole plant or different plant parts such as leaf, fruits, stem, bark, root, flower and seeds Phytochemicals can rapidly respond in synthesis of nanomaterials by reduction of metal salts and due to functional groups, phytochemicals can act as reducing and capping agents. Together with plant materials, nanoparticles show excellent larvicidal activities against mosquito larvae [20]. Furthermore, morphology of the nanoparticles shows different toxicities on mosquito larvae [21].

Nanomaterials serve as nanocarriers to encapsulate and transfer bioactive compounds effectively and precisely. The potency of phytochemical activities relies on maintaining the stability and bioactivity of the active compounds. Environmental factors such as temperature, pH, relative humidity and other biological, physical and chemical factors can influence their activity. Therefore, encapsulation techniques can be introduced to overcome these limitations. Encapsulation emerged to decrease their sensitivity to degradation and can be defined as one or more active compounds immobilized in some form of matrix or walls. Furthermore, they have the ability to control the release of bioactive compounds. [22, 23]. Commonly used inorganic nanomaterials are iron-oxide nanoparticles, mesoporous silica nanoparticles, silver and gold nanoparticles. In addition, biopolymers such as chitosan, xanthun gum and pectin can be used to synthesize nanomaterials for encapsulation. Carbon nanotubes, quantum dots and supercritical fluids can also be used for the encapsulation of bioactive compounds [24].

### Usage of plant-mediated silver nanoparticles against mosquito larvae

Nowadays, silver nanoparticles (AgNPs) are used for synthesis of insecticides due to low toxicity, low cost, eco-friendliness and time efficiency compared with other metal nanoparticles. Furthermore, due to their small spherical shape, they show high potential of larvicidal activities by passing through the cell membrane of larvae cells and then damaging the cellular organelles and functions [25].

Pilaquinga et al. investigated the larvicidal activity of aqueous extract of *Solanum mammosum* fruits and synthesized silver nanoparticles (SmAgNPs) using aqueous extract of this fruit against *Aedes aegypti* larvae. In the synthesis of SmAgNPs, aggregation takes place between AgNO_3_ and fruit extract and results in a colour change from light yellow to brown. Fruit extracts are used as a reducing agent in the synthesis of AgNPs. Different concentrations of *S. mammosum* extract and SmAgNPs were tested against 25 third-instar *Aedes aegypti* larvae and mortality was observed after 24 h. While no mortality was observed in the control group, significant mortality was observed in both *S. mammosum* extract and SmAgNPs. The toxicity of SmAgNPs is much higher than aqueous extract due to the lower LD_50_ value (0.06 and 1631.27 ppm, respectively). As a consequence, *Solanum mammosum* fruit extract modified silver nanoparticles have higher potential in the synthesis of effective larvicides against *Aedes* larvae [6].

The larvicidal efficacy of bark extract of *Holarrhena antidysenterica* and bark extract functionalized silver nanoparticles was investigated against third-instar larvae of *A. aegypti* and *C. quinquefasciatus*. Bark extract functionalized AgNPs were synthesized by incubation of bark extract with silver nitrate solution. The colour change occurred during the reaction from yellow to dark brown due to the reduction of AgNO_3_ to AgNPs by bark extract. The highest larval mortality was exhibited from bark extract modified AgNPs against *A. aegypti* larvae with 5.53 ppm LC_50_ value after 72 h of exposure and no any effects on non-target organisms. Furthermore, bark extracts were prepared using different solvents such as methanol, chloroform, hexane, acetone and water showed moderate larvicidal activity for *A. aegypti* larvae with 71.74, 94.25, 102.25, 618.82, and 353.65 ppm LD_50_ values for respective solvent systems. Compared with larvicidal activity of bark extract alone, bark extract functionalized AgNPs had excellent larvicidal potential in the synthesis of eco-friendly non-toxic larvicides against dengue larvae [4].

A cost-effective, rapid and eco-friendly larvicidal assay was developed using aqueous extract of *Anisomeles indica*-modified silver nanoparticles against malaria, dengue and Japanese encephalitis vectors. The *A. indica*-mediated silver nanoparticles were synthesized by treating plant extract with AgNO_3_ and incubated at room temperature, resulting in a brown colour change in the reaction mixture. Toxicity assessments were conducted separately using a concentration series of aqueous plant crude extract (50, 100, 150, 200 and 250 μg/mL) and a concentration series of synthesized silver nanoparticles (15, 30, 45, 60 and 75 μg/mL). Both *A. indica* leaf extract and AgNPs demonstrated potent larvicidal activities, with effectiveness increasing with concentration in both assays. *A. indica* leaf extract showed 108.98, 120.69 and 131.27 μg/mL LD_50_ values for *A. subpictus*, *A. albopictus* and *C. tritaeniorhynchus* vectors, respectively. Synthesized AgNPs exhibited the highest toxicity, with LD_50_ values of 31.56, 35.21 and 38.08 μg/mL for respective vectors of *A. subpictus*, *A. albopictus*, and *C. tritaeniorhynchus*. Hence, *A. indica*-modified AgNPs hold promise for developing novel larvicidal agents against dengue vectors [15].

*Delphinium denudatum* root extract based bioassay was synthesized as a larvicide against mosquito larvae. They exhibited the reduction and larvicidal property of *D. denudatum* root extract by synthesizing root extract modified silver nanoparticles (DdAgNPs) against second instar larvae of *Aedes aegypti*. *D. denudatum* root extract was mixed and incubated with an AgNO_3_ solution at room temperature in dark conditions and the resulting colour of nanoparticles was yellowish brown. Mortality was observed for bioassay after 24 and 48 h exposure using 10 *Aedes* larvae for each concentration (10, 100 and 1000 ppm) and it was increased with the exposure time. 96 and 9.6 ppm LC_50_ values were obtained from 24 and 48 exposure hours. Therefore, it had a greater larvicidal potential against *Aedes aegypti* larvae and it was less toxic to the environment [7].

Proteins play a predominant role in the formation of the cuticle layer and synthesis of chitin in the insect body, making this phenomenon a potential method for mosquito control. Some proteins found in botanical extracts have the ability to alter the protein composition within insect bodies. The larvicidal efficacy of *Annona reticulata* functionalized silver nanoparticles (Ar-AgNPs) was investigated by exposing the fourth instar larvae of *Aedes aegypti*. *A. reticulata* mediated silver nanoparticles have a specific ability to inhibit the enzymatic activity of mosquitoes by binding to the active sites and blocking them. After exposure to the larvae, the inhibition activity initiated in Ar-AgNPs inhibited the acetylcholine esterase activity and α- and β-carboxylesterase enzyme levels in the mosquito body without developing any resistance. As a result, the bodily functions of mosquitoes are reduced with 20–46% mortalities, 23% larval mortality at 12-h exposure period and 46% larval mortality at 48-h exposure time. This assay was eco-friendly and toxic to the target mosquito larvae but not for non-target species. Larval mortality is low with nanoparticles compared with the mortality of *A. reticulata* plant extract alone [25].

### Usage of plant-mediated gold nanoparticles against mosquito larvae

Green synthesis of gold nanoparticles (AuNPs) also had tremendous attention on mosquito vector control strategies. It facilitated newer, safer and eco-friendly vector control methods against various types of mosquito vectors including *Aedes* larvae. A simple and eco-friendly bioassay was synthesized using *Artemisia vulgaris* leaf extract and AuNPs. Gold nanoparticles were functionalized by *A. vulgaris* leaf extract and leaf extract was mixed with an aqueous solution of gold chloride to synthesize gold nanoparticles. During the reaction, the colour of the solution changed from yellow to purple/red due to the reduction of Au^3+^ to Au^0^ nanoparticles. The resulting AuNPs were tested against third and fourth instar larvae of *Aedes aegypti* to determine the larvicidal efficacy of bioassay at various concentrations (25, 50, 100, 200, 400 ppm). Mortality was concentration dependent; the highest mortality was observed at the highest concentration with the lowest LD_50_ affected by midgut, epithelial cells and cortex. Moreover, the fourth instar larvae exhibited the highest toxic (97.90 and 43.01 ppm LD_50_ after 12 and 24 h, respectively) effect compared with third-instar larvae (156.55 and 62.47 ppm LD_50_ after 12 and 24 h, respectively). Mortality was due to the presence of beta caryophyllene which has insecticidal potential in the synthesis [26].

Moreover, gold nanoparticles were prepared using leaf extract of *Cymbopogon citratus* which acted as reducing and capping material for control of larvae and pupae of *Anopheles stephensi* and *Aedes aegypti*. Mortalities were investigated for both plant extract and plant extract-mediated gold nanoparticles separately. LD_50_ values of gold nanoparticles showed the lowest values (LD_50_ = 18.80- 41.52 ppm) compared with leaf extract of *C. citratus* alone (LD_50_ = 219.32- 471.36 ppm) due to significant differences in larval mortalities that were 77.3% and 56% mortalities for *A. stephensi* and *A. aegypti*, respectively. Thus, *C. citratus* functionalized gold nanoparticles may help to control larvae populations [27].

### Usage of plant-mediated copper nanoparticles against dengue larvae

Various plant extracts contain alkaloids, flavonoids which can act as reducing agents, capable of the reduction of copper ions into copper nanoparticles. Eco-friendly copper nanoparticles (CuNPs) are synthesized using aqueous leaf extract of *Artocarpus heterophyllus* (jackfruit) against *Aedes aegypti* larvae. CuNPs were synthesized by mixing aqueous extract of *A. heterophyllus* with CuSO_4_ at room temperature. A concentration series of nanoparticles (2, 4, 6, 8, 10 mg/mL) were prepared and larvicidal activity was tested with the first to fourth stage larvae of *Aedes aegypti* after a 24-h exposure. Synthesized CuNPs exhibited 100% larval mortality for the highest dose of nanoparticles (10 mg/mL) and no mortality was observed in the control sample. 3.85, 4.24, 4.66, 5.08 mg/mL LD_50_ values were obtained for first to fourth larval stages, respectively, that were very low compared with jackfruit leaf extract alone (21.81, 26.92, 41.38, 55.12 mg/mL for the first to fourth stages of larvae, respectively). *A. heterophyllus* modified CuNPs are very effective as a larvicidal agent for dengue vectors [28].

### Usage of plant-mediated heavy metal nanoparticles against mosquito larvae

Heavy metal nanoparticles also hold significant applications in mosquito control. A study conducted by Hajra et al. synthesized spherical shaped cadmium nanoparticles (CdNPs) which were functionalized by petal extracts of African marigold (*Tagetes* sp.) and rose (*Rosa* sp.). These petal extracts acted as reducing agents to produce cadmium nanoparticles from cadmium ions by mixing cadmium chloride with the flower petal extracts. The resultant marigold extract-mediated nanoparticles showed a yellow solution while rose petal-mediated nanoparticles exhibited a reddish brown solution. When they were exposed to *Aedes albopictus* larvae, 100% mortality was observed in marigold petal extract modified CdNPs, rose petal modified CdNPs showed 98.8% mortality at 10 ppm concentration. Marigold-mediated CdNPs exhibited better performance compared with the rose petal-mediated CdNPs [3].

### Usage of plant extract-mediated metal oxide nanoparticles against dengue larvae

ZnO nanoparticles have excellent multipurpose potential including larvicidal activity as they are easy to prepare, inexpensive, safe, non-toxic and eco-friendly. ZnO nanorods were fabricated using *Myristica fragrans* leaf extract as capping material against the dengue mosquito vector of *Aedes aegypti*. ZnNO_3_ solution was mixed together with *M. fragrans* leaf extract to synthesize ZnO nanorods. A 2, 4, 8, 16 and 32 ppm concentration series was prepared to test the larvicidal activity, which was concentration dependent because 100% larvae mortality occurred at the highest concentration. LD_50_ values were 3.44, 5.25, 8.28 and 14.63 mg/L for first to fourth instar larval stages, respectively. Moreover, the larvicidal activity was tested using 75, 150, 225, 300 and 375 ppm concentrations of *Myristica fragrans* leaf extract and results revealed comparatively high LD_50_ values; 162.03, 194.11, 240.1 and 273.9 ppm for first to fourth larval stages, respectively. ZnO nanorods have excellent larvicidal activity against *Aedes aegypti* [29]*.*

Morphology of the nanoparticle also influences larvicidal activity. Four different structures: star (S), needle (N), plate (P) and cuboidal (C) zinc oxide nanoparticles were synthesized to investigate toxic effects on *Aedes albopictus* and *Anopheles vagus* larvae, which cause dengue and malaria fever. A concentration series of nanoparticles (25, 50, 75 and 100 ppm) was prepared for each morphology and larvicidal activity was tested using second instar larvae of the above vectors at room temperature (25 ℃) and 84 ± 5% relative humidity. The results showed that different morphologies have different larvae mortalities and that all shapes of nanoparticles had larvicidal activities. While star-shaped nanoparticles showed the least LD_50_ value (38.90 ppm), the highest LD_50_ value was obtained from plate-shaped nanoparticles (68.38 ppm) for *Aedes albopictus*. Star-shaped nanoparticles had excellent larvicidal activity compared with others. Moreover, *Anopheles* vectors had the highest toxicity (4.78, 6.51, 13.64, and 10.47 ppm for S, N, P, and C shapes, respectively) compared with *Aedes* vectors (38.90, 47.53, 68.38 and 50.24 ppm for S, N, P, and C shapes, respectively) due to low LD_50_ values for each morphology of nanoparticles [21]. Table 2 presents the larvicidal activities of plant extracts mediated by various nanoparticles discussed in this review.

**Table 2:** Overview on discussed larvicidal activity of different plant extract-mediated nanoparticles in this review

Nanoparticle	Functionalized material plant		Vector	% Mortality	LD_50_ values/ ppm			References
	Plant species	Plant part			24 h	48 h	72 h	
Ag	*Solanum mammosum*	Fruit	*Aedes aegypti*	93.40	0.06	–	–	[6]
	*Holarrhena antidysenterica*	Bark	*Aedes aegypti*	–	–	–	5.53	[4]
	*Anisomeles indica*	Leaf	*Aedes albopictus*	100	35.21	–	–	[15]
	*Delphinium denudatum*	Root	*Aedes aegypti*	100	96	9.6	-	[16]
	*Annona reticulata*	Leaf	*Aedes aegypti*	20–46	–	–	–	[25]
Au	*Artemisia vulgaris*	Leaf	*Aedes aegypti* (3rd stage)	100	62.47	–	–	[26]
			*Aedes. aegypti* (4th stage)		43.01	–	–	
Cd	Marigold (*Tagetes* sp.)	Petal	*Aedes* *albopictus*	100	–	–	–	[3]
	Rose (*Rose* sp.)			98.8	–	–	–	
Cu	*Artocarpus heterophyllus*	Leaf	*A. aegypti* (1st stage)	100	3.85	–	–	[28]
			2nd stage		4.24	–	–	
			3rd stage		4.66	–	–	
			4th stage		5.08	–	–	
ZnO	*Myristica fragrans*	Leaf	*A. aegypti* (1st stage)	100	3.44	–	–	[29]
			2nd stage		5.25	–	–	
			3rd stage		8.28	–	–	
			4th stage		14.13	–	–	

### Usage of plant-mediated polymeric nanoparticles against mosquito larvae

Apart from metallic nanoparticles, polymeric nanoparticles can be used for mosquito control effectively. Chitosan, carboxymethyl cellulose, collagen, dextran, fibrin, gelation, gellan gum, hyaluronic acid, sodium alginate and pectin can be used to synthesize nanoparticles [30]. Amongst them, chitosan is commonly used due to its unique properties. Chitosan is a natural polysaccharide derived from chitin that achieved much attention in the encapsulation of bioactive compounds and essential oils in plants. Chitosan nanoparticles have considerable popularity as a carrier for bioactive compounds due to their non-toxicity, biocompatibility, biodegradability, antimicrobial properties, cost-effectiveness and bio-adhesive properties [31]. In addition, various methods can be employed to synthesize chitosan nanoparticles such as emulsion formation, spray drying, precipitation, ionotropic gelation, coacervation, etc. [32]. Method selection depends on the substances that are going to be encapsulated. Ionic gelation is the conventional method for synthesis of chitosan nanoparticles. Protonated NH_3_^+^ groups of chitosan interact with polyanions in sodium tripolyphosphate (TPP) and undergo crosslinking between monomers. In the presence of secondary metabolites such as alkaloids, they are employed to cross-link chitosan monomers into nanoparticles. The encapsulation of bioactive compounds within the core of gelated chitosan nanoparticles improved the solubility and prevention of bioactive compounds until they reached the target from adverse conditions like lights, microbial infections, etc. Therefore, phytochemical loaded chitosan nanoparticles can be considered as excellent alternatives to synthetic larvicides [33].

The larvicidal potential of chitosan was investigated using extracted chitosan and commercially available chitosan that are extracted from shrimp shells. Chitosan nanoparticles were synthesized using TPP as the crosslinking agent by ionic gelation method. A concentration series (20, 40, 60, 80 and 100 ppm) was prepared and larvicidal activity was tested against third-instar larvae of *Aedes aegypti*. Results showed that higher larval mortality was observed in chitosan nanoparticles from shrimp shells (96%) compared to extracted chitosan (37%) and commercial chitosan (43%) [34].

## Conclusion

This review covers the larvicidal activities of different plant extracts and plant extract-mediated nanoparticles. All plant extracts and green-synthesized nanoparticles containing phytochemicals and essential oils exhibit significant larvicidal activities against dengue vectors of *Aedes aegypti* and *Aedes albopictus*. Synthesized nanomaterials demonstrate superior larvicidal activities compared with plant extract alone. These bioassays are low cost, non-toxic to non-target organisms, environmentally friendly and effective. Effectiveness depends on the phytochemical and essential oil content in the plant, varying with plant species, type of plant part and the solvent used for extraction. Encapsulation processes of bioactive compounds protect their stability and bioactivity.

## Data Availability

Data were obtained from existing published research articles, of which are cited in this review.
